# Nuclear import of dimerized ribosomal protein Rps3 in complex with its chaperone Yar1

**DOI:** 10.1038/srep36714

**Published:** 2016-11-07

**Authors:** Valentin Mitterer, Nadine Gantenbein, Ruth Birner-Gruenberger, Guillaume Murat, Helmut Bergler, Dieter Kressler, Brigitte Pertschy

**Affiliations:** 1 Institut für Molekulare Biowissenschaften, Universität Graz, Humboldtstrasse 50, 8010 Graz, Austria; 2 Institute of Pathology, Research Unit Functional Proteomics and Metabolic Pathways, Medical University of Graz, Stiftingtalstrasse 24, 8010 Graz, Austria; 3 Omics Center Graz, BioTechMed-Graz, Stiftingtalstrasse 24, 8010 Graz, Austria; 4 Unit of Biochemistry, Department of Biology, University of Fribourg, Chemin du Musée 10, CH-1700 Fribourg, Switzerland

## Abstract

After their cytoplasmic synthesis, ribosomal proteins need to be transported into the nucleus, where they assemble with ribosomal RNA into pre-ribosomal particles. Due to their physicochemical properties, they need protection from aggregation on this path. Newly synthesized ribosomal protein Rps3 forms a dimer that is associated with one molecule of its specific chaperone Yar1. Here we report that redundant pathways contribute to the nuclear import of Rps3, with the classical importin α/β pathway (Kap60/Kap95 in yeast) constituting a main import route. The Kap60/Kap95 heterodimer mediates efficient nuclear import of Rps3 by recognition of an N-terminal monopartite nuclear localization signal (NLS). This Rps3-NLS is located directly adjacent to the Yar1-binding site and, upon binding of Kap60 to Rps3, Yar1 is displaced from the ribosomal protein *in vitro*. While Yar1 does not directly interact with Kap60 *in vitro*, affinity purifications of Yar1 and Rps3, however, revealed that Kap60 is present in the Rps3/Yar1 complex *in vivo*. Indeed we could reconstitute such a protein complex containing Rps3 and both Yar1 and Kap60 *in vitro*. Our data suggest that binding of Yar1 to one N-domain and binding of Kap60 to the second N-domain of dimerized Rps3 orchestrates import and protection of the ribosomal protein.

Eukaryotic ribosomes are composed of a small 40S and a large 60S subunit, which consist of ~80 ribosomal proteins (r-proteins) and four ribosomal RNAs (rRNAs), and are highly conserved between yeast and higher eukaryotic species. A highly coordinated and hierarchical assembly of rRNA with r-proteins is required to ensure the accurate production of functional, translation-competent ribosomes. The entire process involves the assistance of a multitude (>200) of transiently acting assembly factors and is one of the major activities of any growing cell. For recent reviews on ribosome biogenesis see refs 1, 2, 3, 4 and 5.

Mammalian and yeast cells need to produce more than one million or ~200,000 ribosomes, respectively, in order to duplicate^6^,^7^. This means that, in yeast, an overall production of ~14 million r-proteins per cell generation is required^7^. These tremendous synthesis rates necessitate an efficient targeting of r-proteins to their ribosomal assembly sites.

In contrast to rRNA, which is synthesized in the nucleolus, r-proteins are translated in the cytoplasm. Most r-proteins are incorporated into ribosomal precursors in the nucleus and consequently need to be imported through nuclear pore complexes (NPCs). A facilitated transport mediated by karyopherins is required to overcome the permeability barrier built up by hydrophobic phenylalanine-glycine (FG) repeat containing nucleoporins within the interior of the central channel of the NPC^8^,^9^,^10^. In the classical import pathway, importin α (Kap60/Srp1 in yeast) functions as a transport adaptor that binds to specific nuclear localization signals (NLS) of cargo proteins as well as to importin β1 (Kap95 in yeast), which interacts with the hydrophobic FG-repeat meshwork^11^,^12^,^13^,^14^. Import of r-proteins was, however, postulated to be mainly performed by the alternative import pathway via karyopherin β4 (Kap123 in yeast), with some redundancy with karyopherin β3 (Pse1/Kap121 in yeast)^15^. These importin β transport receptors do not rely on importin α, but have the ability to recognize their cargoes directly^14^,^16^.

R-proteins are RNA-binding proteins and often contain lysine- and arginine-rich, unfolded extensions that protrude into the interior of the ribosome, where they form critical interactions with rRNA required for the integrity of the ribosomal structure^17^. These positively charged RNA-binding domains, however, cause insolubility when their cognate rRNA binding partners are missing. Besides mediating nuclear import, importins were shown to function as cytoplasmic chaperones that bind to exposed basic domains of r-proteins, which simultaneously comprise rRNA-binding and NLS motifs^18^,^19^. In addition, assembly factors were identified that act as specific holding-chaperones for r-proteins^20^,^21^,^22^,^23^,^24^,^25^,^26^,^27^,^28^. Most of these assembly chaperones were shown to capture the nascent polypeptide chain of their dedicated r-protein clients in a co-translational manner^23^,^24^.

We have previously reported that, prior to its ribosomal integration, the r-protein Rps3 is in a stable complex with its dedicated chaperone Yar1, which serves to keep Rps3 soluble until the r-protein is integrated into ribosome precursors^22^. Yar1 binds to the nascent Rps3 N-domain in the cytoplasm already during its synthesis by the ribosome^23^. Although Yar1 has a cytoplasmic steady-state localization, it transiently enters the nucleus^22^, suggesting that it might accompany Rps3 from the cytoplasm to its nuclear pre-40S assembly site. An N-terminal NLS within Rps3 (Rps3 amino acids 7–10: KKRK) might mediate the proposed co-import of the protein complex. However, the putative NLS is localized directly adjacent to the Yar1-binding site within the first N-terminal α-helix of Rps3; thus, raising the question whether both an importin and Yar1 have simultaneous access to their Rps3 binding region^29^,^30^. Our recent study has revealed that, in complex with Yar1, Rps3 dimerizes with a second Rps3 protein via its C-terminal globular domain^29^. Remarkably, *in vivo*, only one Rps3 N-domain of the dimerized Rps3 is associated with Yar1, while the second Rps3 N-domain is not occupied by the chaperone, resulting in a ternary Rps3/Rps3/Yar1 complex^29^. However, how the formation of this peculiar protein complex is regulated remained unclear.

In this study, we report that the N-terminal Rps3-NLS is recognized by Kap60 and reveal that in contrast to other ribosomal proteins, Rps3 import occurs mainly via the classical importin α/β (Kap60/Kap95) dependent pathway. We show that, *in vitro*, Kap60 and Yar1 compete for the same binding site on Rps3. However, two-step affinity purifications via Yar1-TAP and Rps3-Flag reveal Kap60 to be associated with the ribosome-free Rps3/Rps3/Yar1 complex *in vivo*. Moreover, it is possible to reconstitute a complex *in vitro* that contains Rps3 and both Yar1 and Kap60. Our data suggest that Kap60 binding replaces Yar1 from one Rps3 N-domain of dimerized Rps3, while the second N-domain remains occupied by Yar1. This architecture allows Kap60/Kap95 to promote the coordinated nuclear import of two Rps3 molecules in complex with one Yar1 protein.

## Results

### Kap60/Kap95 mediate Rps3 import via an N-terminal NLS

**Figure 1:**
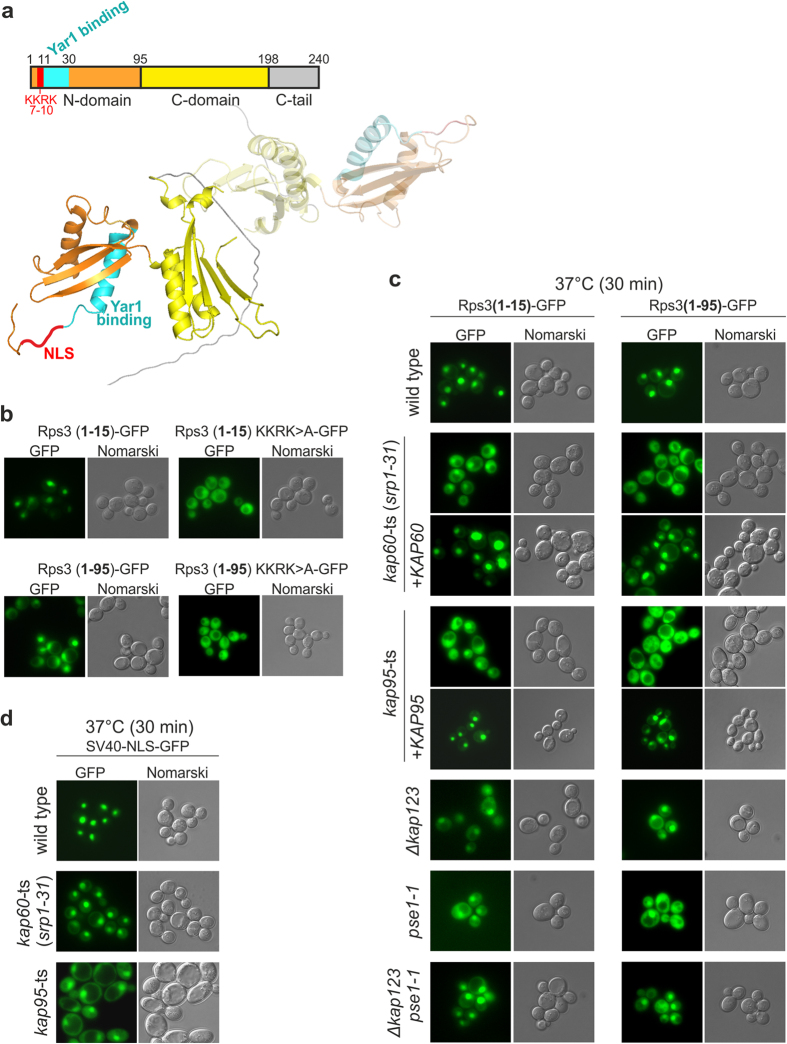
Kap60/Kap95 drive Rps3 nuclear import by recognition of an N-terminal monopartite nuclear localization signal. (**a**) Upper panel: Rps3 is schematically depicted. Lower panel: Rps3 structure extracted from the SAXS model-structure of the Rps3/Yar1 complex^29^. The same colors are used as above. In this complex, Rps3 dimerizes via its C-domain with a second Rps3 copy (depicted in transparent colors). (**b**) N-terminal NLS promotes Rps3 nuclear import. The localization of the indicated Rps3-3xyEGFP reporter constructs was monitored by fluorescence microscopy. (**c**) Nuclear import defects in karyopherin mutant strains. The localization of the indicated Rps3-3xyEGFP reporter-constructs was monitored in wild-type or indicated karyopherin mutant strains grown at 25 °C and shifted for 30 min to 37 °C. Where indicated, the mutant alleles were complemented by plasmid-borne wild-type alleles of the respective karyopherins. (**d**) Localization of a SV40-NLS-3xyEGFP reporter in the indicated strains grown at 25 °C and shifted to 37 °C for 30 min.

Rps3 consists of two globular domains (N- and C-domain), followed by an unstructured C-terminal extension. In the complex with its chaperone Yar1, Rps3 is dimeric (Fig. 1a)^29^. We have previously proposed that four consecutive basic amino acids within the N-terminal Rps3 α-helix (7-KKRK-10) promote its nuclear import (Fig. 1a)^17^,^22^. In line with this, the N-terminal 15 Rps3 amino acids efficiently targeted a 3xyEGFP reporter-construct to the nucleus (Fig. 1b)^22^. Moreover, while full-length Rps3 is incorporated into ribosomes and therefore displays a predominantly cytoplasmic localization^22^, a reporter construct containing the complete Rps3 N-domain (amino acids 1–95) fused to 3xyEGFP localized to the nucleus (Fig. 1b). The nuclear localization of both reporter constructs, however, shifted to the cytoplasm when the four basic N-terminal amino acids were mutated to alanines (KKRK>A constructs), confirming that the KKRK motif is necessary for import (Fig. 1b). In addition, also mutation of only two NLS residues to alanines (K7/K10>A) resulted in a cytoplasmic localization of the reporter constructs (Supplementary Fig. S1a). Hence, the N-terminal KKRK-motif comprises a functional NLS that efficiently targets Rps3 to the nucleus, where it is incorporated into ribosomal precursor particles.

To obtain deeper insights into the regulation of Rps3 nuclear import, we aimed to identify the import receptor(s) responsible for recognition of the N-terminal NLS. We analyzed the localization of the N-terminal Rps3 reporter-constructs (amino acids 1–15 or 1–95 fused to 3xyEGFP) in different karyopherin mutant strains, which were reported to display distinct nuclear import defects^31^,^32^,^33^,^34^,^35^,^36^,^37^,^38^,^39^,^40^. While in some of the tested mutants the nuclear localization of the Rps3-reporter remained unaffected (Supplementary Fig. S1b), we observed a substantial shift to the cytoplasm in temperature-sensitive *kap60* and *kap95* mutant strains (at restrictive temperature, but even when incubated at permissive temperature) (Fig. 1c and Supplementary Fig. S1c). The import of the Rps3 reporter-constructs was restored by providing the respective plasmid-encoded wild-type copies of *KAP60* and *KAP95* (Fig. 1c). Kap60 and Kap95 are the homologues of human importin α and importin β respectively, which were shown to recognize the large T-antigen NLS of Simian-Virus 40 (SV40-NLS)^41^. Since the *kap60* and *kap95* mutant strains displayed at least similarly severe import defects of the Rps3 reporter-constructs as observed for the SV40-NLS fused to 3xyEGFP (Fig. 1d and Supplementary Fig. S1d), we consider the short, monopartite NLS of Rps3 a *bona fide* substrate for the importin α/β-dependent classical import pathway. In addition to *kap60* and *kap95* mutants, nuclear import of the reporter constructs was also impaired in a *kap123Δ* strain and to a smaller extent in a *pse1* mutant strain (Fig. 1c and Supplementary Fig. S1c). These two β-karyopherins were suggested to have partially overlapping protein clients and Kap123 is considered the main importin executing the delivery of r-proteins to their nuclear assembly site^15^. We conclude that Rps3 can be imported into the nucleus via several redundant import routes, including the classical importin α/β-pathway.

**Figure 2:**
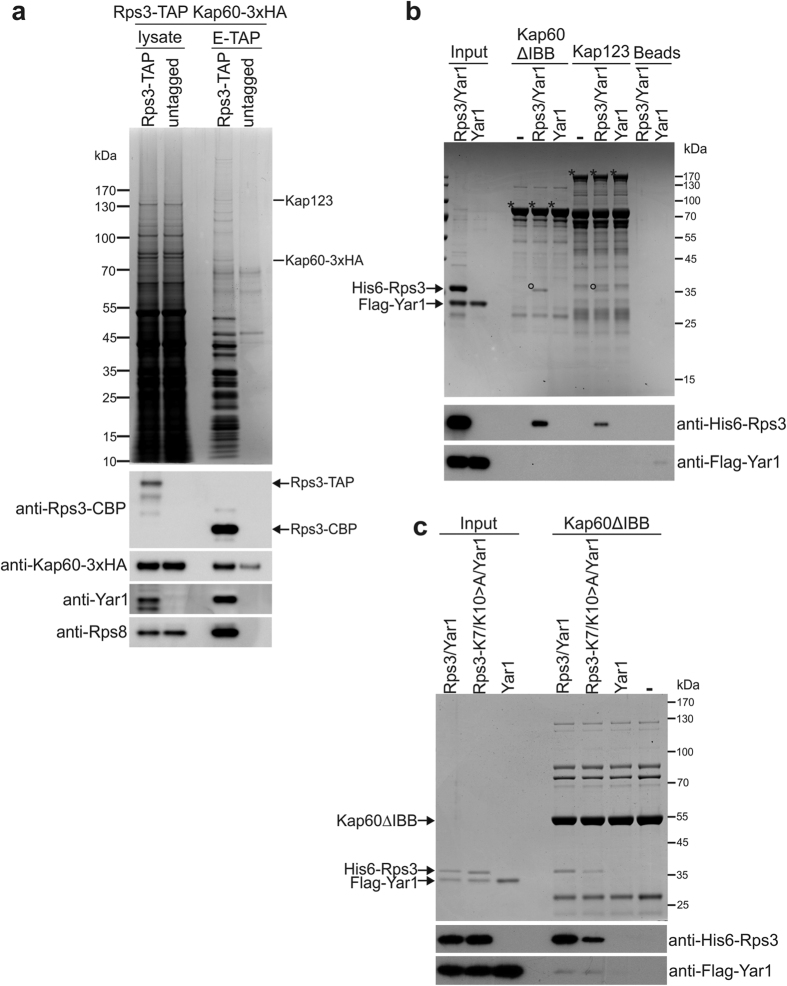
Kap60 and Yar1 compete for Rps3 binding. (**a**) Kap60 co-elutes after Rps3-TAP purification. An *RPS3*-TAP strain carrying a *KAP60*-3xHA fusion was subjected to TAP purification. A *KAP60*-3xHA strain without TAP-tag served as negative control (untagged). Lysates and final eluates (E-TAP) were analyzed by SDS-PAGE followed by Coomassie staining or Western blotting with the indicated antibodies. Note that there is some unspecific recovery of Kap60-3xHA in the eluate of the control strain; however, the protein is clearly enriched in the Rps3-TAP eluate. Kap123: Kap123 was detected in this band by mass spectrometry (Mascot score 211, 11 matched peptides, 12% sequence coverage). Kap60-3xHA: Kap60 was detected in this band by mass spectrometry (Mascot score 85, 4 matched peptides, 10% sequence coverage). (**b**) GST-tagged importins (Kap60ΔIBB or Kap123) were immobilized on glutathione-agarose beads. Truncated Kap60 lacking the N-terminal IBB domain (80 amino acids) was used in order to prevent inhibition of cargo binding by this domain in the absence of Kap95. Beads were incubated with purified, recombinant His6-Rps3/Flag-Yar1 complex or purified Flag-Yar1. Importins and bound material were eluted by boiling. Eluates were analyzed by SDS-PAGE and Coomassie staining or Western blotting with indicated antibodies. Asterisks in the Coomassie stained gel mark the respective importins, circles indicate bound Rps3. (**c**) Disruption of the Rps3-NLS impairs Kap60 binding. GST-tagged Kap60ΔIBB was incubated with His6-Rps3/Flag-Yar1 complex containing wild-type Rps3 or Rps3(K7/K10>A) mutant protein. Kap60ΔIBB was eluted by TEV-cleavage. Eluates were analyzed by SDS-PAGE and Coomassie staining or Western blotting.

In the classical import pathway, importin β1/Kap95 mediates nuclear transport, while importin α/Kap60 engages in cargo binding. To further validate the finding that nuclear import of Rps3 is dependent on Kap60/Kap95, we assessed whether Kap60 is present in a complex with the r-protein *in vivo*. For this purpose, we performed Rps3-TAP purification from a strain in which endogenous Kap60 was C-terminally fused with a 3xHA epitope-tag to allow detection of the importin by Western blot analyzes. Indeed, Kap60 was enriched in the Rps3-TAP eluate after the tandem-affinity purification procedure (Fig. 2a). Furthermore, Kap60 and also Kap123 were detected in the Rps3-TAP eluate by mass spectrometry (Fig. 2a). These results further support the model that a major nuclear import route for Rps3 is via the classical Kap60/Kap95 pathway and that, in addition, Kap123 also contributes to the efficient import of Rps3.

### Kap60 and Yar1 compete for binding to the Rps3 N-domain

We have previously reported that Yar1 functions as a specific chaperone for Rps3 that might accompany the r-protein from the cytoplasm to its ribosome assembly site in the nucleus^22^. Given that the Yar1-binding site and the Rps3-NLS, which likely corresponds to the direct Kap60 interaction surface (see above), are located directly adjacent to each other in the very N-terminal part of Rps3 (Fig. 1a)^29^,^30^, we asked whether binding of Kap60 to the Rps3-NLS allows Yar1 to remain bound to the r-protein and how nuclear co-import of Rps3 in complex with Yar1 is orchestrated. To address this question, we performed *in vitro* binding studies with recombinant GST-tagged importins and His6-Rps3/Flag-Yar1 complex purified from *E. coli*. His6-Rps3 was able to interact to some extent with several, but not all of the tested importins (Supplementary Fig. S2), including Kap123 where significant binding of Rps3 was observed (Fig. 2b and Supplementary Fig. S2). As expected from our *in vivo* data, a very robust interaction was observed between Rps3 and Kap60 (Fig. 2b and Supplementary Fig. S2). Note that a Kap60 truncation lacking its N-terminal importin β-binding site (Kap60ΔIBB) was used in this assay, since in the absence of importin β, the IBB-domain occupies the cargo recognition surface and would therefore prevent substrate binding^42^. The interaction between Rps3 and Kap60 was significantly reduced upon substitution of lysines K7 and K10 within the Rps3-NLS by alanines, confirming the N-terminal NLS as the main target for importin binding (Fig. 2c). Strikingly, our binding studies revealed that Yar1 was not detected bound to Kap60 or any of the other importins, suggesting it was released from the Rps3/Yar1 complex upon binding of Rps3 to the importins (Fig. 2b,c and Supplementary Fig. S2). Since the r-protein was handed over from Yar1 onto Kap60, we conclude that binding of Yar1 and Kap60 to the N-terminal Rps3 α-helix is mutually exclusive.

### Kap60/Kap95 orchestrate nuclear import of the ternary Rps3/Rps3/Yar1 complex

**Figure 3:**
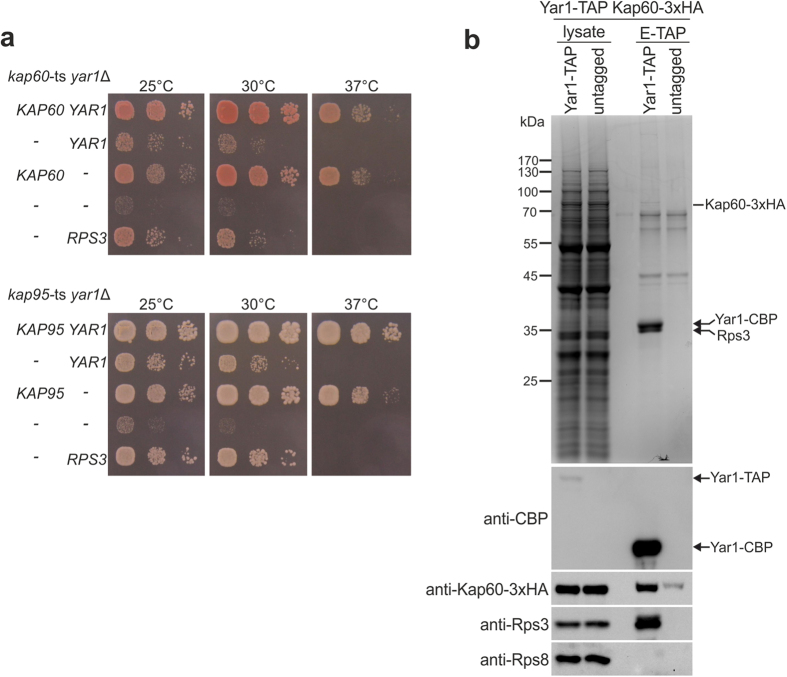
Yar1 and Kap60 are genetically and physically linked. **(a)** Growth defect of a *yar1* deletion strain is enhanced in combination with *kap60-*ts and *kap95-*ts mutant alleles. *kap60-*ts/*yar1*Δ or *kap95-*ts/*yar1*Δ strains were transformed with plasmids harboring the indicated wild-type alleles or empty plasmids (−). Cells were spotted in 10-fold serial dilutions on SD-Leu-Trp plates and incubated at the indicated temperatures. (**b**) Kap60 is co-purified with Yar1-TAP. A *YAR1-*TAP strain carrying a *KAP60*-3xHA fusion was subjected to TAP purification. A strain containing *KAP60*-3xHA but no TAP-tag served as negative control (untagged). Lysates and final eluates (E-TAP) were analyzed by SDS-PAGE followed by Coomassie staining or Western blotting with the indicated antibodies. Kap60-3xHA: Kap60 was detected in this band by mass spectrometry (Mascot score 255, 5 matched peptides, 15% sequence coverage).

We have previously observed that Yar1 transiently enters the nucleus, suggesting that it may protect Rps3 not only in the cytoplasm, but also after nuclear import^22^. However, as shown above, Yar1 was released from Rps3 upon Kap60 binding and was not able to bind to any of the tested importins on its own (Fig. 2b and Supplementary Fig. S2). Notably, we found a genetically enhanced growth defect of yeast strains where the *yar1* deletion was combined with a temperature-sensitive *kap60* or *kap95* allele, as well as a mild genetic interaction when combining the *yar1* and *kap123* deletions, suggesting a functional link between Yar1 and the importins (Fig. 3a and Supplemental Figure S3). These phenotypes were suppressed by increasing the cellular copy number of *RPS3*. Moreover, after Yar1-TAP purification, Kap60-3xHA was recovered in the final eluate in which Rps3 appeared in stoichiometric amounts (Fig. 3b). This was surprising considering that Yar1 and Kap60 directly compete for Rps3 binding and that Yar1 alone was not able to interact with Kap60 directly (Fig. 2b,c and Supplementary Fig. S2). We noticed that the dimeric conformation of Rps3 could allow Rps3/Yar1 co-import by binding of Kap60 to one Rps3 N-domain and Yar1 to the second Rps3 N-domain of the dimer, which would explain the recovery of Kap60 after Yar1-TAP purification.

**Figure 4:**
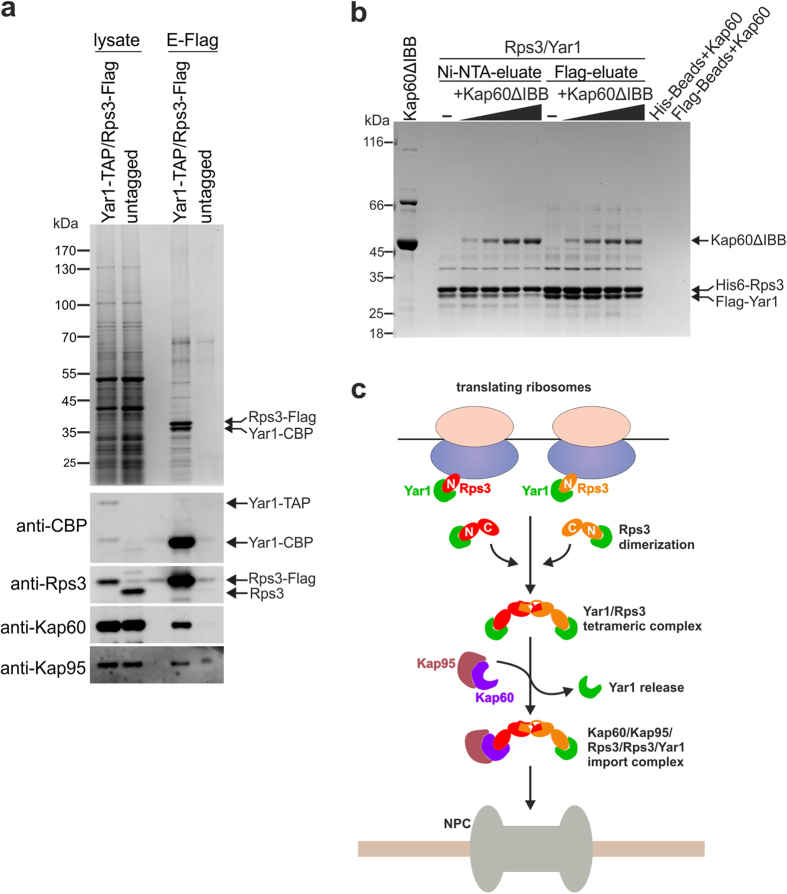
Kap60/Kap95 orchestrate nuclear import of the Rps3/Rps3/Yar1 complex. (**a**) Kap60/Kap95 are bound to the Rps3/Yar1 complex *in vivo*. The cell lysate of a *YAR1*-TAP, *RPS3*-Flag strain was subjected to affinity purification via the protein A tag of Yar1. The obtained TEV (tobacco etch virus) eluate was further purified in a second affinity purification step via Rps3-Flag. As negative control the lysate of an untagged wild-type strain was subjected to the same purification procedure. Lysates and final eluates (E-Flag) were analyzed by SDS-PAGE followed by Coomassie staining or Western blotting with the indicated antibodies. (**b**) Kap60 binds the Rps3/Yar1 complex *in vitro*. His6-Rps3/Flag-Yar1 complex was immobilized via His6-Rps3 on Ni-NTA-agarose beads and incubated with increasing amounts of purified Kap60ΔIBB. After elution of His6-Rps3 (Ni-NTA-eluate), bound material was further subjected to Flag-purification via Flag-Yar1 (Flag-eluate). As negative control, empty Ni-NTA agarose or Flag-agarose beads were incubated with the highest Kap60ΔIBB concentration. The eluates were analyzed by SDS-PAGE and Coomassie staining. (**c**) Model describing the formation of the Yar1/Rps3/Rps3/Kap60/Kap95 import-complex that allows simultaneous targeting of an Rps3 dimer bound to one Yar1 chaperone into the nucleus.

To further test the hypothesis that Kap60 as well as Yar1 occur in the same complex bound to dimerized Rps3, we performed split-tag affinity purification via Yar1-TAP and Rps3-Flag (the TEV eluate of the Yar1-TAP IgG-Sepharose affinity purification was subjected to a second affinity purification via Rps3-Flag to ensure that only complexes containing both Yar1 and Rps3 are purified). Indeed, Kap60 was present in the final eluate of this purification and in addition also Kap95 was detected by western blot analyzes (Fig. 4a). This result confirms that the Kap60/Kap95 heterodimer is bound to the Rps3/Rps3/Yar1 complex *in vivo*. Based on this result, we reasoned that the complete release of Yar1 from Rps3 *in vitro* (Fig. 2b,c and Supplementary Fig. S2) may be due to the high availability of importin in the *in vitro* assays. Consequently, if a Kap60/Rps3/Rps3/Yar1 assembly is in principle possible, it should be feasible to reconstitute such a complex *in vitro* by controlling the amounts of Kap60 added to the Rps3/Yar1 complex. To this end, we immobilized His6-Rps3/Flag-Yar1 complex on Ni-NTA beads via Rps3. We previously observed that, in contrast to the Rps3/Rps3/Yar1 complex occurring *in vivo*, the *in vitro* assembled complex contains not only two Rps3 but also two Yar1 copies^29^. When this *in vitro* purified complex was incubated with increasing amounts of purified Kap60, Yar1 was partially released at high Kap60 concentrations (Ni-NTA eluate, Fig. 4b). Although the reason why Yar1 was not fully released at the highest Kap60 concentrations in this setup is unknown, we speculate that the Rps3 N-domain via which Rps3 is bound to the beads might be less accessible for Kap60 than the second Rps3 N-domain of the dimer. To confirm that both Kap60 and Yar1 are present in the same complex, we subjected the Ni-NTA eluates to a second purification step via Flag-Yar1. Indeed, Flag-Yar1 co-purified not only Rps3, but also Kap60 (Flag-eluate, Fig. 4b). We conclude that in the Yar1-eluate, besides the Yar1/Rps3/Rps3/Yar1 tetramer, a sub-population composed of the proposed Yar1/Rps3/Rps3/Kap60 complex was recovered.

Together, our *in vivo* and *in vitro* results demonstrate that dimeric Rps3 can form a complex containing both Yar1 and importins at the same time. These data are suggestive of a shared import route for Rps3 and Yar1. To address if this is the only possible import pathway for Yar1, we investigated whether Yar1 is able to enter the nucleus also in the absence of Rps3. To this end, we used a strain containing a leptomycin B (LMB) sensitive *crm1* mutant, in which Yar1 is fused to 3xmCherry, allowing trapping of Yar1-3xmCherry in the nucleus upon export inhibition by LMB treatment. Additionally, the strain contained a chromosomal deletion of *RPS3* and a plasmid expressing *RPS3* under the control of the glucose repressible *GAL1* promoter. Surprisingly, Yar1-3xmCherry was detected in the nucleus even after depletion of *RPS3* for two hours, followed by treatment with LMB (Supplementary Figure S4a). This result could be an indication that in the absence of Rps3, Yar1 is also able to enter the nucleus alone. To exclude the possibility that this effect is due to a small residual expression of Rps3 under these conditions, we also wanted to confirm the result with an independent method. Based on the fact that Yar1 interacts with newly synthesized Rps3, we investigated the ability of Yar1-GFP to enter the nucleus after addition of the protein synthesis inhibitor cycloheximide (Supplementary Figure S4b). Also under these conditions, Yar1-GFP accumulated in the nucleus upon export inhibition with LMB. Together, these results indicate that Rps3 is not strictly required for Yar1 import.

As Yar1 was not able to bind to any of the tested importins on its own (Fig. 2b and Supplementary Fig. S2), the import of Yar1 is presumably importin independent. Furthermore, we did not observe interaction of Yar1 with FG-repeat containing nucleoporins (Supplementary Fig. S5). We conclude that in addition to the karyopherin-dependent import pathway for Yar1 bound to dimeric Rps3, Yar1 can presumably also be imported by a different, up to now unknown pathway.

## Discussion

Our results elucidate how the formation of the peculiar Rps3/Yar1 arrangement (with an Rps3 dimer bound to only one Yar1^29^) may be attained by the binding of Kap60. Based on a recent study showing co-translational capturing of the nascent Rps3 polypeptide chain by Yar1^23^, we propose that following Rps3 synthesis, the r-protein dimerizes with a second Yar1-bound Rps3 (Fig. 4c). Subsequent binding of Kap60 to one Rps3-NLS releases one Yar1 molecule from this short-lived complex, while the N-domain of the second Rps3 monomer remains associated with Yar1. An alternative possibility is that Kap60 already associates with Rps3 (thereby releasing Yar1) prior to Rps3 dimerization. It is intriguing that Kap60 does not replace the second copy of Yar1, and we can only speculate how the formation of the peculiar Kap60/Rps3/Rps3/Yar1 complex is regulated. One possibility is that formation of the complex containing both Yar1 and Kap60 is simply more likely due to a higher availability of Yar1, which, in contrast to Kap60, has no substrates other than Rps3. Moreover, Kap60 binding likely results in the rapid import of the complex, hence limiting the time for a second importin to get access to the second Rps3 copy. In this scenario, the formation of complexes containing two Kap60 molecules would also be possible, resulting in import of a subpopulation of newly synthesized Rps3 by Kap60 directly, without encountering Yar1. Nevertheless, the lower probability of this event would ensure that the majority of complexes have both Kap60 and Yar1 bound. Alternatively, cells might utilize more active mechanisms to favor the formation of the Kap60/Rps3/Rps3/Yar1 complex. For example, binding of Kap60 to one Rps3 may not be sterically compatible with dimerization with a second Rps3/Kap60 complex, or the *in vivo* Rps3 dimer may have an intrinsic asymmetry favoring binding of Kap60 to one Rps3 and Yar1 to the second Rps3 molecule.

After capturing the Rps3 N-domain, Kap60/Kap95 promote the simultaneous and Yar1-escorted nuclear transport of dimeric Rps3, which may be beneficial for the cell as it targets two Rps3 molecules to ribosome assembly with only one import event. In addition, alternative import routes, e.g. via Kap123, presumably also contribute to Rps3 import. However, as Yar1 was also released upon binding of Rps3 to these alternative importins (Fig. 2b and Supplementary Fig. S2), all these routes may utilize a similar mechanism as Kap60 and mediate import of dimeric Rps3 in complex with one Yar1 molecule. We observed that disruption of the N-terminal Rps3-NLS by mutating lysines K7 and K10 to alanines (due to insolubility of the protein we were not able to purify sufficient amounts of an Rps3 variant in which all four basic amino acids of the NLS were mutated to alanines) resulted in a significant reduction but not complete abolishment of Kap60 binding (Fig. 2c). Possibly, the remaining positively charged amino acids of the NLS (K8 and R9) are sufficient to mediate this residual interaction. Alternatively, a second NLS-motif within Rps3 might mediate this weak interaction with the importin. In line with this, alternative import routes using additional NLS sequences have been proposed also for other r-proteins. In the case of Rpl4, five distinct NLSs have been identified that may contribute to an efficient nuclear import of the protein^24^. Another example is the synchronized nuclear targeting of Rpl5 and Rpl11, which occurs via the NLS of the adaptor-protein Syo1, which is recognized by Kap104^43^. Since Syo1 is a non-essential protein, an alternative import pathway for Rpl5 and Rpl11 has to exist. However, as an alternative NLS within Rps3 would presumably not overlap with the Yar1 binding site (Rps3 amino acids 11–30), no competition between Yar1 and importin binding would be expected. Since our *in vitro* binding studies showed that Yar1 did not bind in complex with Rps3 to any of the tested importins (Fig. 2b and Supplementary Fig. S2), an alternative NLS would therefore provide only a very minor contribution to Rps3 import.

Notably, Yar1 is also able to enter the nucleus in the absence of newly synthesized Rps3 (Supplementary Fig. S4). As Yar1 neither interacted with the tested importins nor with the tested FG-repeat containing nucleoporins, some other transport pathway may account for Yar1 import in the absence of Rps3. Interestingly, a recent study suggests that a subgroup of ankyrin-repeat proteins can utilize a novel import pathway in which they form a complex together with Ran-GDP and the Ran-GDP import factor Ntf2^44^. Yar1 does not strictly follow the consensus of ankyrin-repeat proteins with this ability, however it cannot be excluded that it still has sufficient affinity for Ran-GDP to utilize this pathway. Another possibility would be that Yar1 interacts transiently with some other protein, which is able to mediate import of Yar1 in the absence of Rps3. Future studies will have to elucidate the actual mechanism underlying Yar1 import and to address whether this so far unknown pathway is also able to recognize Yar1 bound to Rps3.

We have recently shown that the slow-growth phenotype entailed by disruption of the N-terminal Rps3 NLS/KKRK-motif does not result from the inhibition of Rps3 import but from the failure to establish crucial interactions with Rps20 and rRNA, suggesting that Rps3 import via the N-terminal NLS is not absolutely essential^29^. This points towards an alternative, so far unknown import pathway for Rps3, or might be an indication that Rps3 can alternatively also assemble at the stage of cytoplasmic 40S precursors. The examination of these possibilities will be an interesting subject for future studies.

## Experimental Procedures

### Strains and plasmids

*S. cerevisiae* strains used in this study are W303 derivatives generated by established gene disruption and tagging methods and are listed in Table S1. Yeast and *E. coli* plasmids were constructed using standard recombinant DNA techniques and are listed in Tables S2 and S3.

### Fluorescence microscopy

Rps3-truncations and SV40-NLS were C-terminally fused to a triple yEGFP (3xyEGFP) tag and expressed from plasmids under transcriptional control of the *ADH1* promoter. Live yeast cells were imaged by fluorescence microscopy using either a Zeiss Axioskop microscope or an Olympus BX54 microscope. For the experiments in Supplementary Figure S4, when indicated, Leptomycin B (LMB) was added to a final concentration of 200 ng/μl. In Supplementary Figure S4a cells were grown in SD medium containing 2% raffinose and 0.2% galactose. *RPS3* expression was turned off by shifting the cells for 2 h to SD medium containing glucose. In Supplementary Figure S4b, when indicated, cells were incubated for 20 min in presence of 50 μg/ml cycloheximide prior to LMB addition.

### Tandem Affinity Purifications (TAP)

Rps3-TAP, Yar1-TAP, and Yar1-TAP/Rps3-Flag strains were each cultured in 2L YPD medium at 25 °C to an OD_600_ of ~1.8. The affinity purification steps were carried out as described previously^29^,^45^. Samples were analyzed on 4–12% (Rps3-TAP, Yar1-TAP/Rps3-Flag) or 12% (Yar1-TAP) SDS-polyacrylamide gels (NuPAGE, Invitrogen) followed by Colloidal Coomassie staining (Invitrogen) or Western blotting with the indicated antibodies.

### *In vitro* pull down assays

Expression plasmids were transformed into an *E. coli* BL21 (DE3) Rosetta Star strain. For expression of His6-Rps3/Flag-Yar1 complex, Flag-Yar1, His6-Yar1, His6-Arx1, or GST-tagged nucleoporins, cells were cultured in LB-medium at 30 °C to an OD_600_ of 0.3–0.4. For expression of GST-tagged importins, cells were cultured in high-yield protein expression medium^46^ containing 10 g/l yeast extract, 10 g/l glucose, 5 g/l diammonium hydrogen phosphate, 4,5 g/l potassium dihydrogen phosphate, 2,5 g/l disodium hydrogen phosphate, 1,5 g/l magnesium sulfate, 1,2 g/l ammonium ferric citrate, 0,2 g/l ammonium chloride, 1 ml/l trace element solution SL-6 (containing 100 mg/l zinc sulfate, 30 mg/l manganese dichloride, 300 mg/l boric acid, 200 mg/l cobaltous chloride, 10 mg/l cupric chloride, 20  mg/l nickel dichloride, 30 mg/l sodium molybdate) at 30 °C to an OD_600_ of ~0.8. Protein expression was induced with 0.3 mM isopropyl-β-D-thiogalactoside (IPTG) and cultures were shifted to 16 °C for 20 h. Subsequent protein purification was performed as described previously^29^. Cells were lysed by sonication in lysis buffer containing 50 mM Tris (pH 7.5), 150 mM NaCl, 1 mM DTT, 0.5 mM PMSF, HP protease inhibitor cocktail (Serva), 1 mg/ml lysozyme (for proteins purified via His6-tag 40 mM imidazole was added). His6-tagged proteins were bound to Ni-NTA agarose (Qiagen) and eluted with 300 mM imidazole. Flag-Yar1 was bound to anti-Flag agarose (Sigma) and eluted with Flag-peptide (Sigma). GST-tagged proteins were immobilized on glutathione-agarose beads (Sigma) and incubated with indicated eluates of purified proteins. Beads were washed 5 times with 5 volumes of lysis buffer (w/o protease inhibitors and lysozyme) and subsequently either suspended in SDS sample buffer and eluted by boiling at 95 °C (Fig. 2b and Supplementary Fig. S2), suspended in lysis buffer and eluted by TEV-protease cleavage for 1 h at room temperature (Fig. 2c), or eluted with 20 mM reduced glutathione (Sigma) (Supplementary Fig. S5). Eluates were analyzed on 12% or 4–12% SDS-polyacrylamide gels (Invitrogen) and Coomassie staining or Western blotting.

For the *in vitro* competition experiment in Fig. 4b, His6-Rps3/Flag-Yar1 were expressed as described above and the protein complex was immobilized to Ni-NTA agarose (Qiagen). After washing steps, the Ni-NTA beads were incubated with increasing amounts of purified Kap60ΔIBB for 1 h at 4 °C (recombinant GST-Kap60ΔIBB was expressed and purified as described above. The protein was eluted with TEV protease, thereby removing the GST-tag). Beads were washed 6 times, removing unbound material, and subsequently His6-Rps3 and interacting proteins were eluted for 20 min with 300 mM imidazole. Next, the eluates were incubated for 1 h at 4 °C with anti-Flag agarose beads (Sigma), which were then washed 6 times to remove unbound material. Flag-Yar1 and interacting proteins were eluted with Flag-peptide for 1h at 4 °C. As negative control, empty Ni-NTA agarose or Flag-agarose beads (without His6-Rps3/Flag-Yar1 complex) were incubated with the highest Kap60ΔIBB concentration. Imidazole- and Flag-eluates were analyzed on 4–12% SDS-polyacrylamide gels (Invitrogen) and Coomassie staining.

### Western Blotting

Western blot analysis was performed using the following antibodies: anti-Yar1 antibody (1:5,000)^22^, anti-Rps3 antibody (1:30,000, provided by Matthias Seedorf), anti-Rps8 antibody (1:5,000, provided by Giorgio Dieci), anti-CBP antibody (1:4,000, Millipore), secondary anti-rabbit horseradish peroxidase-conjugated antibody (1:15,000, Sigma), anti-importin α antibody (1:200, Santa Cruz Biotechnology, sc-32681), anti-Kap95 antibody (1:200, Santa Cruz Biotechnology, sc-27053), secondary anti-goat horseradish peroxidase-conjugated antibody (1:12,000, Sigma), horseradish peroxidase-conjugated anti-His6 antibody (1:10,000, Sigma), horseradish peroxidase-conjugated anti-Flag antibody (1:10,000, Sigma), horseradish peroxidase-conjugated anti-HA antibody (1:5,000, Roche).

### Mass spectrometry

Excised gel bands were reduced, alkylated and digested with Promega modified trypsin. Digests were analyzed by LC-MS/MS. Peptide extracts were dissolved in 0.1% formic acid, desalted online and separated by nano-RP-HPLC using a 140 min gradient. Peptides were analyzed in a Bruker maXis 2 Q-TOF mass spectrometer operated in positive ion mode, applying alternating full scan MS (m/z 200 to 2000) and MS/MS by collision induced dissociation of the 17 most intense peaks with dynamic exclusion enabled. The LC-MS/MS data were analyzed by searching the yeast SwissProt database with Bruker ProteinScape 4 and Mascot 2.3 (1% FDR using Percolator, min. one rank 1 peptide with min. Mascot ion score 10 and max. 10 ppm precursor mass error required for protein identification).

## Additional Information

**How to cite this article**: Mitterer, V. *et al*. Nuclear import of dimerized ribosomal protein Rps3 in complex with its chaperone Yar1. *Sci. Rep.* **6**, 36714; doi: 10.1038/srep36714 (2016).

**Publisher’s note**: Springer Nature remains neutral with regard to jurisdictional claims in published maps and institutional affiliations.

## Supplementary Material

Supplementary Information
