# Supplementary material for: Nuclear import of dimerized ribosomal protein Rps3 in complex with its chaperone Yar1
Source: Sci Rep. 2016 Nov 7;6:36714. doi: 10.1038/srep36714 (PMC5098186; doi:10.1038/srep36714)
Supplement: Supplementary Information [file srep36714-s1.pdf]

Supplementary Information

Nuclear import of dimerized ribosomal protein Rps3 in complex with its chaperone Yar1

Valentin Mitterer[1], Nadine Gantenbein[1], Ruth Birner-Gruenberger[2,3], Guillaume Murat[4], Helmut Bergler[1], Dieter Kressler[4], Brigitte Pertschy[1,*]

[1] Institut für Molekulare Biowissenschaften, Universität Graz, Humboldtstrasse 50, 8010 Graz, Austria

[2] Institute of Pathology, Research Unit Functional Proteomics and Metabolic Pathways, Medical University of Graz, Stiftingtalstrasse 24, 8010 Graz

[3] Omics Center Graz, BioTechMed-Graz, Stiftingtalstrasse 24, 8010 Graz

[4] Unit of Biochemistry, Department of Biology, University of Fribourg, Chemin du Musée 10, CH-1700 Fribourg, Switzerland

* Correspondence: brigitte.pertschy@uni-graz.at (B.P.)

Supplementary Figure 1

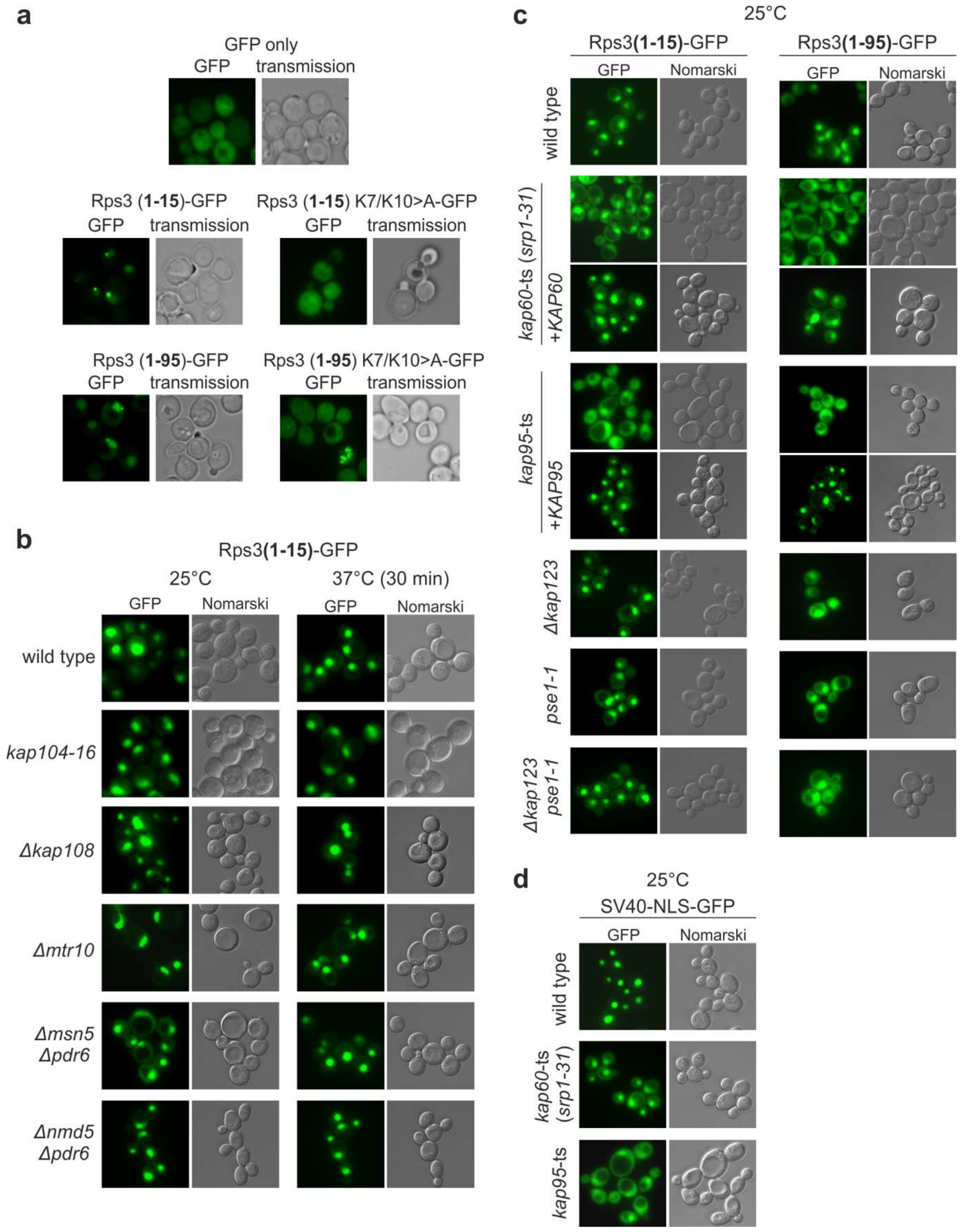

Supplementary Figure S1. Kap60/Kap95, Kap123 and Pse1 are required for efficient nuclear import of Rps3. The localization of the indicated N-terminal Rps3 reporter constructs (**a**), (**b**) and (**c**) or SV-40NLS (**d**) fused to 3xyEGFP was assessed by

fluorescence microscopy in W303 wild-type or the indicated karyopherin mutant strains after incubation at the indicated temperatures. (**a**) 3xyEGFP alone was used as control and displayed predominantly cytoplasmic localization.

Supplementary Figure 2

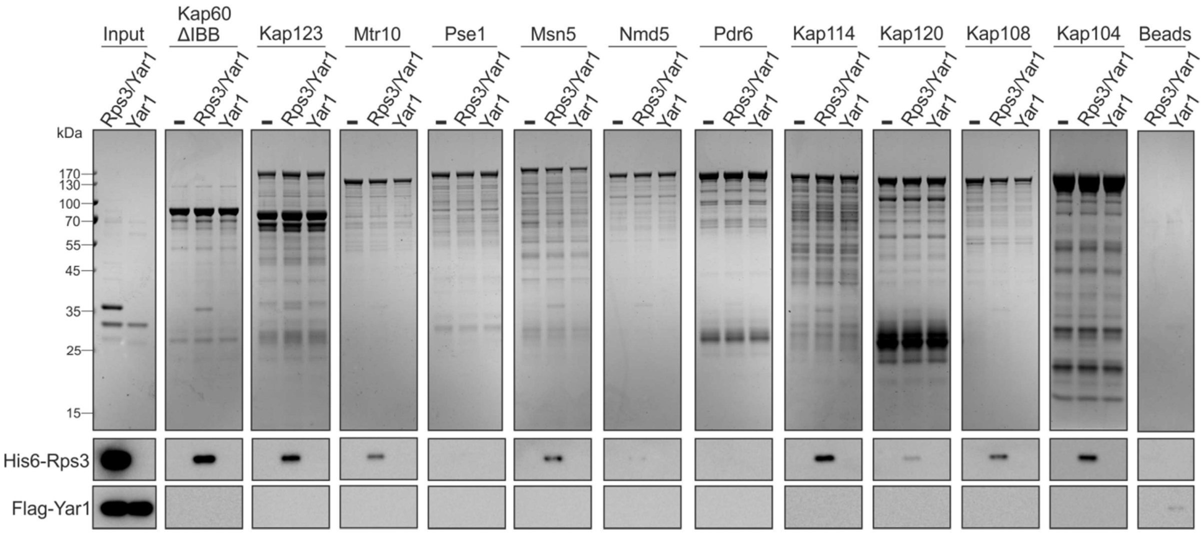

Supplementary Figure S2. Rps3 is transferred from the Rps3/Yar1 complex onto importins. The indicated GST-tagged importins were expressed in *E. coli*, immobilized on glutathione-agarose beads and subsequently incubated with purified His6-Rps3/Flag-Yar1 complex, purified Flag-Yar1 or buffer (-). As negative control, empty glutathione-agarose beads were incubated with the His6-Rps3/Flag-Yar1 complex or Flag-Yar1 (beads). After subsequent washing steps, bound material was eluted and analyzed by SDS-PAGE and Coomassie staining or Western blotting with the indicated antibodies. Notably, Yar1 was not detected bound to any of the tested importins, also not in the samples where Rps3-binding was observed.

Supplementary Figure 3

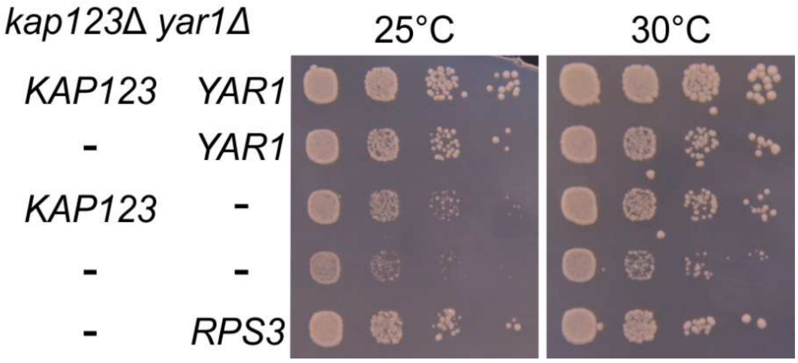

Supplementary Figure S3. Deletion of *KAP123* slightly enhances the growth defect of a *yar1* deletion strain. A *kap123*Δ *yar1*Δ strain was transformed with plasmids harboring the indicated wild-type alleles or empty plasmids (-). Cells were spotted in 10-fold serial dilutions on SD-Ura-Leu plates and incubated at the indicated temperatures for 3 days.

Supplementary Figure 4

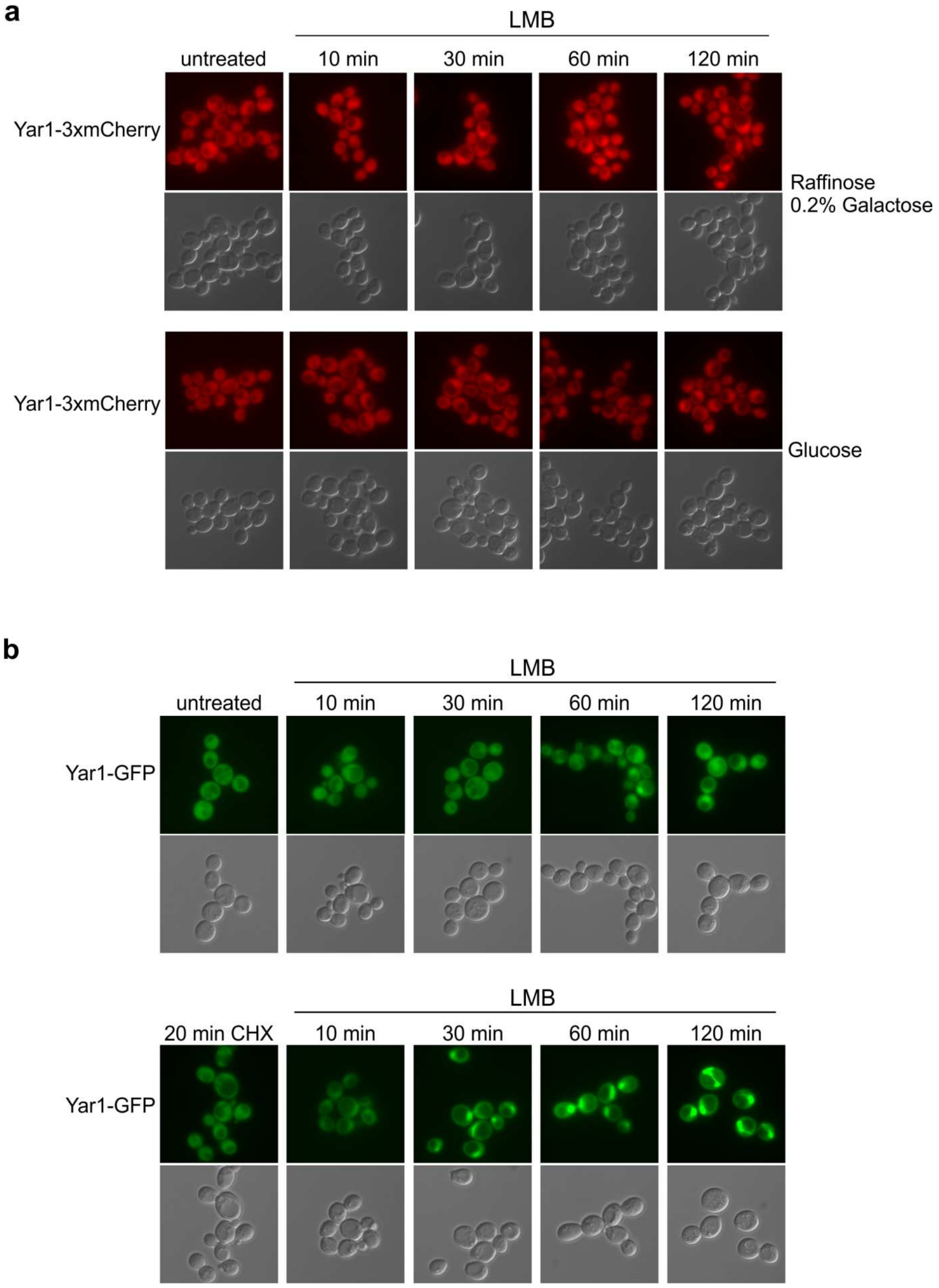

Supplementary Figure S4. Yar1 enters the nucleus after Rps3 depletion or inhibition of protein synthesis. (a) A 3xmCherry tag was C-terminally fused to the *YAR1* gene locus in a leptomycin B (LMB)-sensitive *crm1*T539C *rps3*Δ mutant strain in which plasmid-encoded *RPS3* was expressed under the control of a *GAL1* promoter The localization of the Yar1-3xmCherry fusion protein was assessed under conditions allowing Rps3 expression (medium containing 2% raffinose and 0.2% galactose as carbon source) (upper panel) or after depletion of Rps3 for 2h in medium containing glucose as carbon source (lower panel). The Yar1-3xmCherry localization was examined in untreated cells or after addition of LMB for the indicated time. **(b)** A LMB-sensitive *crm1*T539C *yar1*Δ strain was transformed with a plasmid encoding a Yar1-eGFP fusion protein. The localization of Yar1-eGFP was assessed in untreated cells or in cells treated with LMB for the indicated time (upper panel). The localization of Yar1-eGFP was assessed after incubation for 20 min with cycloheximide (CHX) prior to LMB addition (lower panel).

Supplementary Figure 5

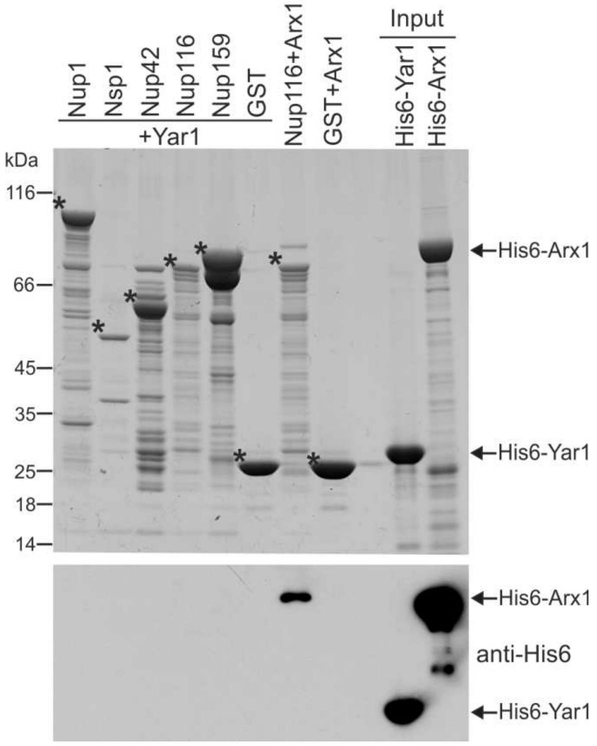

Supplementary Figure S5. Yar1 does not interact with nucleoporins. The indicated GST-tagged nucleoporins (or truncations thereof; see Table S3) were immobilized on glutathione-agarose beads and incubated with purified His6-Yar1. As positive control the Nup116 FG-repeat fragment was incubated with purified His6-Arx1 (pre-60S export factor). Bound material was eluted in buffer containing 20 mM reduced glutathione and samples were subsequently analyzed by SDS-PAGE and Coomassie staining or Western blotting. Asterisks indicate the respective bait proteins.

Table S1. ***S. cerevisiae*** **strains**

**Name**	**Genotype**	**Source**
W303	*ade2-1, his3-11, 15, leu2-3,112, trp1-1, ura3-1, can1-100*	[1]
srp1-31/kap60ts	W303 *MAT***a** *srp1-31*	[2]
kap95ts	*MATα leu2 his3 trp1 ura3 rsl1-4*	Ed Hurt lab, backcross from PSY1103[3] with W303
Δ*kap123* (PSY967)	W303 *MAT*α *kap123::HIS3*	[4]
pse1-1 (PSY1201)	W303 *MAT***a** *pse1-1*	[4]
Δ*kap123 pse1-1* (PSY1042)	W303 *MAT***a** *pse1-1 kap123::HIS3*	[4]
Δ*sxm1* (PSY1200)	W303 *MAT***a** *sxm1::HIS3*	[4]
Δ*mtr10*	W303 *MAT*α *mtr10::HIS3*	[5]
Δ*msn5* Δ*pdr6*	W303 *MAT***a** *pdr6::HIS3 msn5::TRP1*	Ed Hurt lab
Δ*nmd5* Δ*pdr6*	W303 *MAT***a** *pdr6::HIS3 nmd5::HIS3*	Ed Hurt lab
RPS3-TAP *KAP60*-3xHA	W303 *MAT***a** *RPS3*-TAP::natNT2 *KAP60*-3xHA:: *HIS3*MX6	this study
YAR1-TAP *KAP60*-3xHA	W303 *MAT***a** *YAR1*-TAP::natNT2 *KAP60*-3xHA:: *HIS3*MX6	this study
KAP60-3xHA	W303 *MAT***a** *KAP60*-3xHA:: *HIS3*MX6	this study
YAR1-TAP *RPS3*-Flag	W303 *MAT***a** *YAR1*-TAP::HIS3MX6 *RPS3*-Flag::natNT2	this study
srp1-31 Δ*yar1*	W303 *MAT***a** *srp1-31* yar1::*HIS3*MX6	this study (*YAR1* knockout in *srp1-31*)
*kap95*ts Δ*yar1*	*MATα leu2 his3 trp1 ura3 rsl1-4 yar1*::*HIS3*MX6	this study (*YAR1* knockout in *kap95* ts)
KAP104 shuffle	*MAT*α *kap104*::natNT2 *ade3*::kanMX4 [pRS316-*KAP104*]	[6]
Δ*kap123* Δ*yar1*	W303 *MAT***a** *ade3::kanMX4 yar1*::natNT2 *kap123*::*HIS3*MX6	this study
*crm1*T539C *YAR1*-3xmCherry pGAL111-*RPS3*	W303 *MAT***a** *ADE2 crm1*T539C::kanMX4 *YAR1-3xmCherry*::*hphNT1 rps3*::natNT2 [pGAL111-RPS3]	this study
Δ*crm1* Δ*yar1* pRS315-crm1T539C	W303 MAT**a** *crm1*::kanMX *yar1*::natNT2 [pRS315-crm1T539C]	this study (based on MNY8 strain from Neville and Rosbash [7])

Table S2. *S. cerevisiae* plasmids

Name	Relevant Information	Source
p*ADH*111-*RPS3*(1-15)-(GA)$_5$-3xyEGFP	CEN, *LEU2*, P*ADH1*, T*ADH1,* C-terminal (GA)$_5$3xyEGFP	[8]
p*ADH*111-*RPS3*(1-15.KKRK>A)-(GA)$_5$-3xyEGFP	CEN, *LEU2*, P*ADH1*, T*ADH1,* C-terminal (GA)$_5$3xyEGFP	this study
p*ADH*111-*RPS3*(1-15.K7/K10>A)-(GA)$_5$-3xyEGFP	CEN, *LEU2*, P*ADH1*, T*ADH1,* C-terminal (GA)$_5$3xyEGFP	this study
p*ADH*111-*RPS3*(1-95)-(GA)$_5$-3xyEGFP	CEN, *LEU2*, P*ADH1*, T*ADH1,* C-terminal (GA)$_5$3xyEGFP	this study
p*ADH*111-*RPS3*(1-95.KKRK>A)-(GA)$_5$-3xyEGFP	CEN, *LEU2*, P*ADH1*, T*ADH1,* C-terminal (GA)$_5$3xyEGFP	this study
p*ADH*111-*RPS3*(1-95.K7/K10>A)-(GA)$_5$-3xyEGFP	CEN, *LEU2*, P*ADH1*, T*ADH1,* C-terminal (GA)$_5$3xyEGFP	this study
p*ADH*111- (GA)5-3xyEGFP	CEN, *LEU2*, P*ADH1*, T*ADH1,* C-terminal (GA)$_5$3xyEGFP	[8]
p*ADH*111-SV40-NLS-(GA)$_5$-3xyEGFP	CEN, *LEU2*, P*ADH1*, T*ADH1,* C-terminal (GA)$_5$3xyEGFP	[8]
pRS314-*KAP60*	CEN, *URA3*	[9]
pRS314-*KAP95*	CEN, *URA3*	Ed Hurt lab, subcloned from [10]
pRS315-*YAR1*	CEN, *LEU2*, P*YAR1*, T*YAR1*	[8]
pRS315-*RPS3*	CEN, *LEU2*, P*RPS3*, T*RPS3*	[8]
pRS314-*kap104-16*	CEN, *TRP1*, P*KAP104*, T*KAP104*	[11]
YCplac22-*KAP123*	CEN, *TRP1*, P*KAP123*, T*KAP123*	this study
p*GAL*111-*RPS3*	CEN, *LEU2*, P*GAL1*, T*ADH1*	this study
pRS316-*YAR1*-EGFP	CEN, *URA3*, P*YAR1*, T*ADH1*	[8]

P and T denote promoter and terminator, respectively.

Table S3. *E. coli* expression plasmids

Name	Relevant Information	Source
pETDuet-1-His6-Rps3/Flag-Yar1	amp^r, T7 promoter/*lac* operator	this study
pETDuet-1-His6-Rps3(K7/K10>A)/Flag-Yar1	amp^r, T7 promoter/*lac* operator	this study
pETDuet-1-Flag-Yar1	amp^r, T7 promoter/*lac* operator	[12]
pETDuet-1-His6-Yar1	amp^r, T7 promoter/*lac* operator	[8]
pProEx-GST-TEV-Kap60ΔIBB (Kap60 amino acids 81-542)	amp^r, TRC promoter/*lac* operator	this study
pGEX-4TEV-*KAP123*	amp^r, TAC promoter/*lac* operator	[13]
pGEX-4TEV-*PSE1*	amp^r, TAC promoter/*lac* operator	[13]
pGEX-4TEV-*KAP104*	amp^r, TAC promoter/*lac* operator	[14]
pGEX-4T-*SXM1*	amp^r, TAC promoter/*lac* operator	[15]
pGEX-4T-*NMD5*	amp^r, TAC promoter/*lac* operator	[15]
pGEX-5G-*KAP120*	amp^r, TAC promoter/*lac* operator	[15]
pGEX-4T-*KAP114*	amp^r, TAC promoter/*lac* operator	[15]
pGEX-4T-*PDR6*	amp^r, TAC promoter/*lac* operator	[15]
pGEX-4TEV-*MTR10*	amp^r, TAC promoter/*lac* operator	[15]
pGEX-4T-*MSN5*	amp^r, TAC promoter/*lac* operator	[15]
pGEX-Nup1 (amino acids 332-1076)	amp^r, TAC promoter/*lac* operator	[16]
pGEX-Nsp1-C (amino acids 591-823)	kan^r, TAC promoter/*lac* operator	[17]
pGEX-Nup42	amp^r, TAC promoter/*lac* operator	[16]
pGEX-Nup116 (amino acids 165-715)	amp^r, TAC promoter/*lac* operator	[16]
pGEX-Nup159 (amino acids 457-900)	amp^r, TAC promoter/*lac* operator	[18]
pET32a-Arx1	amp^r, T7 promoter/*lac* operator	this study

Supplementary References

1. Thomas, B. J. & Rothstein, R. Elevated recombination rates in transcriptionally active DNA. *Cell* **56,** 619–630 (1989).
2. Loeb, J. D. *et al.* The yeast nuclear import receptor is required for mitosis. *Proc. Natl. Acad. Sci. U. S. A.* **92,** 7647–7651 (1995).
3. Goffin, L. *et al.* The unfolded protein response transducer Ire1p contains a nuclear localization sequence recognized by multiple beta importins. *Mol. Biol. Cell* **17,** 5309–5323 (2006).
4. Seedorf, M. & Silver, P. A. Importin/karyopherin protein family members required for mRNA export from the nucleus. *Proc. Natl. Acad. Sci. U. S. A.* **94,** 8590–8595 (1997).
5. Senger, B. *et al.* Mtr10p functions as a nuclear import receptor for the mRNA-binding protein Npl3p. *EMBO J.* **17,** 2196–2207 (1998).
6. Pillet, B. *et al.* The Dedicated Chaperone Acl4 Escorts Ribosomal Protein Rpl4 to Its Nuclear Pre-60S Assembly Site. *PLoS Genet.* **11,** e1005565 (2015).
7. Neville, M. & Rosbash, M. The NES-Crm1p export pathway is not a major mRNA export route in Saccharomyces cerevisiae. *EMBO J.* **18,** 3746–3756 (1999).
8. Koch, B. *et al.* Yar1 protects the ribosomal protein Rps3 from aggregation. *J. Biol. Chem.* **287,** 21806–21815 (2012).
9. Yano, R., Oakes, M. L., Tabb, M. M. & Nomura, M. Yeast Srp1p has homology to armadillo/plakoglobin/beta-catenin and participates in apparently multiple nuclear functions including the maintenance of the nucleolar structure. *Proc. Natl. Acad. Sci. U. S. A.* **91,** 6880–6884 (1994).
10. Koepp, D. M., Wong, D. H., Corbett, A. H. & Silver, P. A. Dynamic localization of the nuclear import receptor and its interactions with transport factors. *J. Cell Biol.* **133,** 1163–1176 (1996).
11. Aitchison, J. D., Blobel, G. & Rout, M. P. Kap104p: a karyopherin involved in the nuclear transport of messenger RNA binding proteins. *Science* **274,** 624–627 (1996).

12. Mitterer, V. *et al.* Sequential domain assembly of ribosomal protein S3 drives 40S subunit maturation. *Nat. Commun.* **7,** 10336 (2016).

13. Fries, T. *et al.* A novel conserved nuclear localization signal is recognized by a group of yeast importins. *J. Biol. Chem.* **282,** 19292–19301 (2007).

14. Maurer, P. *et al.* The nuclear export receptor Xpo1p forms distinct complexes with NES transport substrates and the yeast Ran binding protein 1 (Yrb1p). *Mol. Biol. Cell* **12,** 539–549 (2001).

15. Caesar, S., Greiner, M. & Schlenstedt, G. Kap120 functions as a nuclear import receptor for ribosome assembly factor Rpf1 in yeast. *Mol. Cell. Biol.* **26,** 3170–3180 (2006).

16. Allen, N. P., Huang, L., Burlingame, A. & Rexach, M. Proteomic analysis of nucleoporin interacting proteins. *J. Biol. Chem.* **276,** 29268–29274 (2001).

17. Bailer, S. M., Balduf, C. & Hurt, E. The Nsp1p carboxy-terminal domain is organized into functionally distinct coiled-coil regions required for assembly of nucleoporin subcomplexes and nucleocytoplasmic transport. *Mol. Cell. Biol.* **21,** 7944–7955 (2001).

18. Strässer, K., Bassler, J. & Hurt, E. Binding of the Mex67p/Mtr2p heterodimer to FXFG, GLFG, and FG repeat nucleoporins is essential for nuclear mRNA export. *J. Cell Biol.* **150,** 695–706 (2000).
